# Entrepreneurial leadership, nurses’ proactive work behavior, and career adaptability: a structural equation model

**DOI:** 10.1186/s12912-024-01804-4

**Published:** 2024-02-27

**Authors:** Nadia Hassan Ali Awad, Heba Ahmed Hamza Zabady, Gehan Galal Elbialy, Heba Mohamed Al-anwer Ali Ashour

**Affiliations:** Nursing Administration Department, Faculty of Nursing, Alexandria University, Alexandria, Egypt

**Keywords:** Entrepreneurial leadership, Nurses, Proactive work behavior, Career adaptability, A structural equation model

## Abstract

**Background:**

Healthcare organizations with practitioners who exhibit proactive work behavior and career adaptability acquire a competitive advantage in the face of many adversities. Entrepreneurial leadership (EL) is a new leadership approach that has a huge impact on followers’ behavior, although research into its theory and empirical evidence is still in its infancy.

**Methods:**

A non-probability convenience sample methodology (*n* = 450) was utilized to choose study participants, who were equally dispersed among the two private hospitals in Alexandria. A cross-sectional study was carried out in all departments of the hospitals, which were chosen at random using a simple random procedure. Three validated scales were used in this study to measure the study variables and establish a structural equation model.

**Results:**

The result of this study revealed that nurses perceived moderate mean scores of all variables; entrepreneurial leadership (140.84 ± 11.94), proactive work behavior (46.02 ± 5.85), and career adaptability (85.55 ± 10.35). In addition, the structured equation model revealed a goodness fit index and presents that entrepreneurial leadership significantly affects nurses’ proactive work behavior with an estimated β of 0.555, coefficient of regression C.R. of 4.006, at *P* value < 0.001. Also, it significantly affects career adaptability with an estimated β of .834, a coefficient of regression C.R. of 3.491 at *P* value < 0.001.

**Conclusions:**

The developed structural equation model confirmed the significant impact of entrepreneurial leadership (EL) on nurses’ proactive work behavior (PWB) and career adaptability (CA)”. Therefore, this study offers important implications for nurse managers, staff nurses, hospital human resources management practice, and academics.

## Background

Aging, illness prevalence, pandemics, rising clinical expenses, scarce resources, quickly developing and disruptive technology, and economic pressures all pose threats to the sustainability and competitive advantage of healthcare corporations. At the same time, healthcare professionals, particularly nurses, are expanding their clinical responsibilities and changing their careers to deliver a variety of high-quality, efficient services while fostering a positive public perception. By enacting a unique kind of leadership called entrepreneurial leadership (EL), which varies from traditional managerial leadership. This style highlights a leader’s qualities and actions that may foster entrepreneurial behaviors. Those behaviors like spotting and seizing opportunities, encouraging proactive work habits, and encouraging career flexibility among nurses to achieve favorable career outcomes and organizational triumph in the healthcare industry [1–4].

### Theoretical framework

**Fig. 1:**
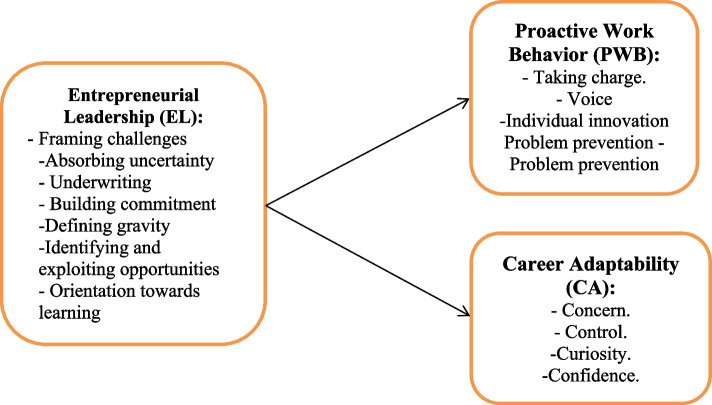
Proposed researchers’ study framework

The research paradigm is elucidated by Bandura’s social cognitive theory (SCT), which offers a theoretical framework for grasping the effect of entrepreneurial leadership on nurses’ proactive work practices and career adaptability. According to SCT, an individual’s beliefs, deeds, and perceptions impact how they engage with behavior. Social structures and environmental influences often play a role in the development and change of human beliefs and cognitive capacities in this interaction between an individual and their surroundings. An individual’s conduct influences the features of their environment, which in turn influences their behavior, constituting the ultimate interaction between the environment and behavior [5]. The proposed conceptual framework in this study is built using this theoretical framework as a guideline (Fig. 1).

### Entrepreneurial leadership (EL)

EL is the conduct of a leader who captivates followers with a compelling vision, gains their loyalty, and dedicates themselves to identifying and generating strategic value. EL combines the management of start-ups’ leadership with their entrepreneurial drive, enabling new businesses to create the required diversity and navigate a complex, dynamic environment by identifying avenues for growth, taking calculated risks, pointing out directions, and putting ideas into action. By their distinctive offerings, leaders methodically and interpersonally lead, facilitate, and mobilize a group of followers toward achieving exceptional performance and fulfilling the corporate goal. Nurse Managers have the potential to invigorate work activities by formulating a novel vision that can profoundly impact nurses’ capacity for career flexibility and proactive work habits [6–8].

EL comprises seven key components. Framing the challenge is the ability of entrepreneurial leaders to set extraordinarily lofty goals and standards for the competence of people and their work. Absorbing uncertainty refers to an entrepreneur’s ability to take risks, foresee potential future developments, accept responsibility, form a vision, see the possibilities, and instill confidence in the staff. Underwriting is a way for an entrepreneurial leader to show their engagement in interpersonal negotiations and use their persuasive skills to persuade others to accept their ideas while also providing direction and support to followers [9, 10].

Building commitment involves inspiring followers, fortifying the group’s dedication to achievement, and fostering a sense of cohesion and collaboration among teammates*.* Defining gravity is the capacity to develop unity and cohesion via mutual understanding and consensus over the goals that need to be accomplished. Identifying and exploiting opportunities is defined as the entrepreneurial leader’s capacity to assess the variables that influence the probability of success or failure and make informed decisions. Orientation towards learning is an attitude toward learning that emphasizes development programs, being current with developing trends, and having a sharp sense of where to find relevant information [9, 10].

It has been shown through studies that EL enhances both individual and organizational performance. It acts as a catalyst to boost an organization’s competitiveness by empowering it to successfully negotiate environmental and crisis-related challenges and achieve sustainable growth, holistic care, and preservation. Additionally, it had a major impact on the development of strategies and tactics that promote creativity and opportunity recognition as well as unrestricted behavior (proactive, inventive, and organizational citizenship behavior) by modifying, responding to, promoting, and utilizing opportunities in the workplace to improve enactment [6–12].

### Proactive work behavior (PWB)

PWB entails anticipation, planning, and action to influence the future. It is also characterized as predictive, self-generated activities aimed at the future to improve the situation at hand or one’s personal qualities. Furthermore, it was clarified that an individual’s aspirational conduct, which is typified by a proactive strategy incorporating a proactive mentality, eagerness, and inventiveness, can initiate a shift in the current circumstances or generate new chances inside the workplace. PWB differs from a reactive and passive mindset that entails waiting for uncontrollable outside events to occur [13–15].

Four elements are integrated into PWB. Taking charge is an intentional, self-directed endeavor started by nurses who are actively working to bring about good change inside the enterprise. Voice is the intention of attaining a favorable result. Individual innovation is a practice that comprises creating and utilizing advantageous modern thoughts to support the organization’s strategic durability and productive work. Problem prevention involves identifying the root of the issue and taking action to stop it from happening again soon. These proactive, independent preventative efforts are crucial to preventing workplace problems from recurring [16, 17].

PWB is essential for fostering career adaptability (CA) and attaining positive adaption outcomes, such as task competency, individual inventiveness, lower turnover, improved performance, and enhanced productivity that benefits the business. It helps nurses deliberately become experts in their work environment, especially when faced with change and uncertainty. This can help them feel competent in their roles. To anticipate upcoming trends and chances for gaining a competitive edge, it also functions as issue-selling and proactive external milieu analysis [18, 19]..

### Career adaptability (CA)

CA is a psychosocial foundation and a concoction of attitudes, abilities, and behaviors that help people manage difficult situations in their line of work, such as disappointments, traumatic experiences, and upcoming or current job changes. It’s a capacity to handle the predictable duties involved in getting ready for and doing a job, as well as the erratic alterations brought on by changes in the workplace and its surroundings. Additionally, it enables nurses to make career decisions and handle workplace difficulties by understanding both themselves and their occupation. As a result, nurses must be prepared to learn novel talents and adopt new working styles to deal with advances [20, 21].

Concern, control, curiosity, and confidence are the four hallmarks that comprise career adaptability. The concern is a future-oriented dimension of CA and comprises the mental process of imagining prospective career paths and devising strategies to accomplish professional objectives. Control demonstrates how much nurses endorse self-control and accept responsibility for their future occupational success, establishing a feeling of autonomy and precision and facilitating an efficient performance of professional and developmental duties. Curiosity refers to the exploration of one’s potential professions. It arises from a curious attitude toward future work opportunities as well as a willingness to investigate scenarios when faced with career changes. Confidence is described as feelings of self-confidence and nurses’ belief in their capacities to pursue professional goals and aspirations in the face of threats [22].

CA improves career management and provides a better understanding of work-related concerns. It has a favorable impact on occupational and work outcomes, including job success, job performance, well-being, employability, entrepreneurial intentions, and professional fulfillment, as well as lower job stress and attrition [23, 24].

### Significance of study

Nurses’ performance has been influenced by many factors, including their usage of the internet, telecommunications, and highly revolutionary technology like artificial intelligence. Furthermore, because of the current COVID-19 outbreak, nurses have faced increased work pressures, a work-life imbalance, and a nursing shortage, according to the ICN study report 2023. The nurse shortage has increased dramatically from 30.6 million in 2019 when the epidemic began. Furthermore, COVID-19 has altered the dynamic structure of contemporary organizations, forcing both practitioners and scholars to focus on establishing work behaviors that are change-oriented, future-focused, self-starting, and career-adapted. These variables are reflected in scholars’ findings and documented high levels of nurses’ turnover intentions, low percentage of career adaptability, and proactive work behaviors among nurses [25–30].

Among several contextual factors, EL is an emerging leadership style that is still in its embryonic stages of empirical and theoretical development and has a considerable impact on the behaviors of followers [31]. Despite previous research demonstrating the importance of entrepreneurial leadership, less is known about the influence of nurse managers’ EL behaviors on nurses’ PWB and CA. Therefore, this study addresses this empirical knowledge gap theoretically and empirically and gives further exploration of the importance of EL for nurse managers in improving PWB and the CA of nurses.

## Method

### Research question and hypotheses

What is the relationship between EL, nurses’ PWB, and CA?

Null H0: There is no significant relationship between EL, nurses’ PWB, and CA.

Alternative H1: EL as an independent variable will significantly correlate with both nurses’ PWB and CA.

#### The aim of the study

This study is directed at developing a structure equation model for testing the relationship between entrepreneurial leadership as an independent variable and nurses’ proactive work behavior and career adaptability as a dependent variable.

#### Research design and setting

Based on the proposed framework, a cross-sectional and correlational study was done using three validated scales to measure the study variables. This study was conducted in all departments at two private hospitals in Alexandria that were selected randomly through a simple random method after conducting a sampling frame of all private hospital names that were similar in the types of service provided and slightly had the same bed capacity and number of nurses.

#### Participant

In this study, a non-probability convenience sampling technique of (*n* = 450) nurses was utilized to collect data, with an equal number from each selected hospital (*n* = 225). To be considered, participants had to meet the following criteria: (a) have more than 3 months of experience; and (b) willingly participate.

#### Instruments

##### Tool (I): entrepreneurial leadership questionnaire (ELQ)

This tool was developed by Bagheri and Harrison (2020) and has strong internal consistency in previous studies to examine nurses’ perceptions of their nurse managers’ EL practices [10, 32]. It is made up of 40 items with high internal reliability (α =0.98) that are organized into seven dimensions: framing the challenge (5 items), underwriting (5 items), defining gravity (5 items), orientation towards learning (5 items), absorbing uncertainty (4 items), building commitment (6 items), and opportunity identification and exploitation (10 items). The replies were graded on a 5-point Likert scale, with 1 being strongly disagreeing and 5 being strongly agreeing. The overall mean score varied from 40 to 200, with a score of (40- 93) indicating low nurses’ perception of their nurse managers’ EL practices, a score of (94 - less than 147) indicating moderate perception, and a score of (147- 200) indicating high perception.

##### Tool (II): proactive work behavior scale (PWBS)

Parker and Collins (2010) constructed this tool, which has shown good internal consistency in earlier research to assess the frequency of nurses’ PWB [33, 34]. The survey consists of 13 highly reliable questions (with a reliability score of α = 0.93) and is divided into four categories: taking responsibility (3 questions), voice (4 questions), individual innovation (3 questions), and problem prevention (3 questions). The responses were graded on a 5-point scale, ranging from (1) extremely infrequent to (5) very regular. The overall average score ranged from 13 to 65, with scores between 13 and 30 indicating a low frequency of perceived PWB (psychological well-being) among nurses, scores between 31 and less than 48 indicating moderate frequency, and scores between 48 and 65 indicating high frequency of perceived PWB among nurses.

##### Tool (III): career adaptability scale (CAS)

Porfeli and Savickas (2012) settled this scale and demonstrated excellent internal consistency in earlier research to assess nurses’ perception of CA [22, 35]. It is made up of 24 elements with good internal dependability (α = 0.90). Concern (6 items), control (6 items), curiosity (6 items), and confidence (6 items) were the four dimensions. The replies were graded on a 5-point Likert scale, with 1 being strongly disagreeing and 5 being strongly agreeing. The total mean score varied from (24 to 120), with the score from (24 to less than 56) indicating a low level of CA, the score from (56 to less than 88) indicating a moderate level of CA, and the score from (88 to 120) indicating a high degree of CA.

Furthermore, the study subject’s demographic characteristics and work-related data sheet, which include questions on age, sex, marital status, educational background, and length of job experience.

### Validity and reliability

Cronbach’s alpha correlation coefficient was utilized to assess the internal consistency of the study instruments. The results confirmed that the three instruments were reliable. Five academics in the field evaluated the translation’s fluency and content validity by translating tools into Arabic for Egyptian cultural relevance. As a result, several elements have been changed to improve clarity. Language experts then translated the tools back into English. The researchers and jury members assessed the back-translations to verify accuracy and reduce any threats to the research’s validity. We also conducted a test run on 45 nurses (10%) who were not study participants to verify that the tools were clear and applicable, as well as to estimate the amount of time it would take to complete the questions. Considering the pilot research findings, no amendments have been made to the final tools.

### Data collection

Having formal approval from the governing body for the designated environment enabled data collection to take place. Data was collected after the researchers described the purpose of the study and obtained subjects’ consent by delivering questionnaires personally to study participants. Each nurse completed the questionnaires in around 30 minutes. Data was collected over 4 months, from February to May of 2023. During data collection, the researchers tried to overcome the common method biases by ensuring confidentiality and anonymity of replies as they notified the participants that the information they provide will be safely stored, combined, and utilized exclusively for the study. Additionally, the researchers used various response forms to gather the required data, create surveys with items arranged in a random sequence, and gather data at various intervals.

### Statistical analysis

Cronbach’s alpha was used to assess the tools’ dependability. The participant’s demographic data and study variables were analyzed using frequency and percent; the study variables were described using a mean score and standard deviation. Pearson’s correlation was used to study the associations between EL, PWB, and CA. AMOS Ver. 23 was utilized to investigate the impact of entrepreneurial leadership on nurses’ proactive work behavior and career adaptability through structural equation modeling (SEM). The model fit indices included the evaluation of; χ2/df = Chi Square/degree of freedom, CFI = Comparative fit index; IFI = Incremental Fit Index; RMSEA = Root Mean Square Error of Approximation; NFI = Normed fit index; RFI = Radio Frequency Interference; NNFI (Non-Normed Fit Index); SRMR (Standardized Root Mean Square Residual); GFI (Goodness of Fit Index); AGFI (Adjusted Goodness of Fit); TLI (Tucker-Lewis Index). The model can be judged for its fitness based on Schermelleh et al., (2003) [36] as follows;

**Table:** 

Fit parameter	Good fit	Acceptable fit
χ2	0 ≤ χ2 ≤ 2df	2df < χ2 ≤ 3df
p value	.05 < *p* ≤ 1.00	0.01 ≤ *p* ≤ 0.05
χ2/df	0 ≤ χ2/df ≤ 2	2 < χ2/df ≤ 3
RMSEA	0 ≤ RMSEA ≤ .05	0.05 < RMSEA ≤0.08
SRMR	0 ≤ SRMR ≤ .05	0.05 < SRMR ≤ .10
NFI	0.95 ≤ NFI ≤ 1.00	0.90 ≤ NFI < 0.95
RFI	0.95 ≤ RFI ≤ 1.00	0.90 ≤ RFI < 0.95
TLI	0.95 ≤ TLI ≤ 1.00	0.90 ≤ TLI < 0.95
NNFI	0.97 ≤ NNFI ≤1.00	0.95 ≤ NNFI < 0.97
CFI	0.97 ≤ CFI ≤ 1.00	0.95 ≤ CFI < 0.97
GFI	0.95 ≤ GFI ≤ 1.00	0.90 ≤ GFI < 0.95
AGFI	0.90 ≤ AGFI ≤1.00	0.85 ≤ AGFI < 0.90

## Results

### Background characteristics of the participants

The mean age of the staff nurses was 34.20 ± 7.72 years. Half of them were in the age group of 30–40 years old. Most of them (92%) were female, and more than two-thirds (66.7%) were married. Concerning educational qualifications, 43.5% of them had a bachelor’s degree in nursing science. The mean years of experience were 9.12 ± 5.24 years, with 30.9% of them having 5 to less than 10 years of experience.

Table 1 declared that nurses perceived a moderate mean score of overall EL (140.84 ± 11.94), which represented a moderate mean score of its related dimensions: framing the challenges (18.35 ± 1.77), absorbing uncertainty (14.08 ± 2.05), underwriting (19.25 ± 2.03), building commitment (17.17 ± 2.20), defining gravity (20.37 ± 1.80), opportunity identification and exploitation (32.17 ± 4.44), and orientation towards learning (19.45 ± 2.39).

**Table 1:** Mean score and standard deviation of nurses’ perceptions of EL of their nurse manager

EL dimensions	Mean ± SD
Framing the challenge	18.35 ± 1.77
Absorbing uncertainty	14.08 ± 2.05
Underwriting	19.25 ± 2.03
Building commitment	17.17 ± 2.20
Defining Gravity	20.37 ± 1.80
Opportunity identification and exploitation	32.17 ± 4.44
Orientation towards learning	19.45 ± 2.39
**Total EL**	**140.84 ± 11.94**

Table 2 illustrates that nurses perceived a moderate frequency mean score of overall PWB (46.02 ± 5.85), which represented a moderate mean score of its related dimensions: problem prevention (10.63 ± 1.58), individual innovation (9.24 ± 1.54), voice (14.72 ± 2.02), and taking charge (11.43 ± 1.81).

**Table 2:** Mean score and standard deviation of nurses’ perceptions of the frequency of their PWB

PWB dimensions	Mean ± SD
Problem prevention	10.63 ± 1.58
Individual innovation	9.24 ± 1.54
Voice	14.72 ± 2.02
Taking charge	11.43 ± 1.81
**Total PWB**	46.02 ± 5.85

Table 3 showed that nurses perceived a moderate mean score of overall CA (85.55 ± 10.35) which represented in moderate mean score of its related dimensions; concern (19.18 ± 3.13), control (23.30 ± 3.28), curiosity (21.00 ± 3.03) and confidence (22.07 ± 2.85).

**Table 3:** Mean score and standard deviation of nurses’ perceptions of their level of CA

CA dimensions	Mean ± SD
Concern	19.18 ± 3.13
Control	23.30 ± 3.28
Curiosity	21.00 ± 3.03
Confidence	22.07 ± 2.85
**Total Career Adaptability**	**85.55 ± 10.35**

Table 4 clarifies that there was a statistically significant positive moderate correlation between EL and PWB, EL and CA, and CA and PWB where (*r* = 0.449, *p* = 0.000**), (*r* = 0.359, *p* = 0.000**), (*r* = 0.319, *p* = 0.002**).

**Table 4:** Correlation Matrix between EL, PWB, and CA

Variables		EL	PWB	CA
EL	r	1	0.449	0.359
	P		0.000**	0.000**
PWB	r		1	0.319
	P			0.000**

**Fig. 2:**
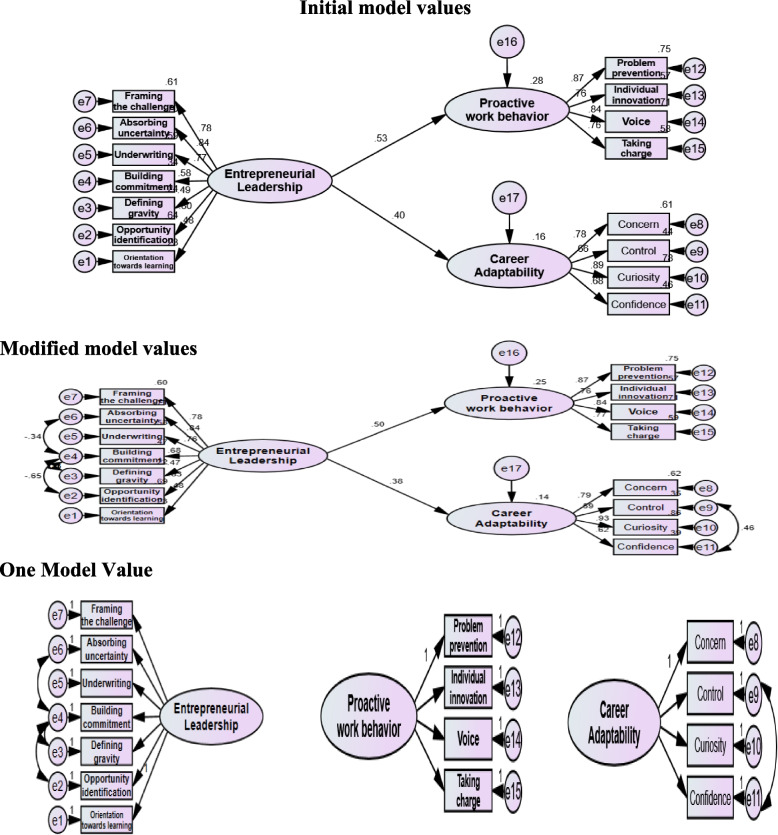
Structure equation modeling of EL as an independent variable and its impact on nurses’ PWB and CA

Table 5 and Fig. 2 Testing on construction models was done to confirm the suggested research hypothesis. According to the test results, the goodness of fit index is composed of the following values: CFI = 0.928; IFI = 0.930; RMSEA = 0.082; TLI = 0.910; RMR = 0.276; SRMR = 0.090; X^2^ = 134.789; *P =* 0.000; df = 84 and χ2/df = 1.605. However, the values of NFI = 0.834; RFI = 0.793; GFI = 0.845; AGFI = 0.779; and NNFI = 0.7925 were slightly less than the acceptable level. It can be claimed that the structural the majority of model’s parameters revealed a good fit index. So, it can be said that this model presents that EL has a significant effect on nurses’ PWB with an estimated β of 0.785, coefficient of regression C.R. of 3.415, at *P* value < 0.001. Also, it significantly affects CA with an estimated β of 0.997, a coefficient of regression C.R. of 2.72 at *P* value < 0.001.

**Table 5:** Structure equation modeling of EL as an independent variable and its impact on nurses’ PWB and CA

Study variables			Initial Model Values				Modified Model Values			
			Estimate	S.E.	C.R.	P	Estimate	S.E.	C.R.	P
PWB	<−--	EL	0.848	0.242	3.498	***	0.785	0.228	3.451	***
CA	<−--	EL	1.048	0.372	2.818	0.005	0.997	0.367	2.72	0.007
Orientation towards learning	<−--	EL	1				1			
Opportunity identification and exploitation	<−--	EL	4.651	1.035	4.495	***	4.787	1.013	4.726	***
Defining gravity	<−--	EL	1.339	0.38	3.521	***	1.272	0.362	3.515	***
Building commitment	<−--	EL	1.679	0.434	3.869	***	1.979	0.469	4.221	***
Underwriting	<−--	EL	2.149	0.485	4.428	***	2.114	0.464	4.559	***
Absorbing uncertainty	<−--	EL	2.009	0.44	4.562	***	2.016	0.431	4.678	***
Framing the challenge	<−--	EL	1.87	0.42	4.449	***	1.851	0.403	4.59	***
Concern	<−--	CA	1				1			
Control	<−--	CA	0.739	0.118	6.287	***	0.662	0.117	5.662	***
Curiosity	<−--	CA	1.128	0.139	8.116	***	1.176	0.158	7.456	***
Confidence	<−--	CA	0.789	0.122	6.483	***	0.718	0.123	5.852	***
Problem prevention	<−--	PWB	1				1			
Individual innovation	<−--	PWB	0.918	0.11	8.347	***	0.917	0.111	8.259	***
Voice	<−--	PWB	1.501	0.155	9.655	***	1.5	0.154	9.755	***
Taking charge	<−--	PWB	0.938	0.111	8.43	***	0.943	0.112	8.448	***
			**Initial Model Value** Model X2; significance 180.787; 0.000, df = 88. χ2/df = 2.054 Model fit parameters; CFI; IFI; RMSEA, NFI; RFI; GFI; AGFI; TLI; NNFI;SRMR (0.869; 0.872; 0.108, 0.777; 0.734; 0.789; 0.713; 0.843; 0.734; 0.092).				**Modified Model Values** Model X2; significance 134.789; 0.000, df = 84. χ2/df = 1.605 Model fit parameters; CFI; IFI; RMSEA, NFI; RFI; GFI; AGFI; TLI; NNFI;SRMR (0.928; 0.930; 0.082, 0.834; 0.793; 0.845; 0.779; 0.910; 0.7925;0.090).			

χ2 Discrepancy Chi Square; χ2/df = Chi Square/degree of freedom, *CFI*  Comparative fit index, *IFI*  Incremental Fit Index, *RMSEA*  Root Mean Square Error of Approximation, *NFI*  Normed fit index, *RFI*  Radio Frequency Interference, *NNFI* Non-Normed Fit Index, *SRMR*  Standardized Root Mean Square Residual, *GFI* Goodness of Fit Index, *AGFI* Adjusted Goodness of Fit, *TLI* Tucker-Lewis Index

Table 6 Initial Model Values, Modified Model Values, and One Factor Model Values of EL, CA, and PWB demonstrate the validity and reliability of the study variables. Based on the test result of Confirmatory Factor Analysis (CFA) on the construct of EL, it is seen that the value of λ (loading factor) in every dimension is > 0.5. It means that all the indicators in the dimensions have been valid (first order). Likewise, the value of CR is ≥0.70, and VE is≥0.50. Thus, it can be concluded that all the indicators used in this study have good validity and reliability values. Considering CA, the value of λ (loading factor) in every dimension is > 0.5. It means that all the indicators in the dimensions have been valid (first order). Likewise, the value of CR is ≥0.70, and VE is ≥0.50. Thus, it can be concluded that all the indicators used in this study have good validity and reliability values. As regards PWB, the value of λ (loading factor) in every dimension is > 0.5. It means that all the indicators in the dimensions have been valid (first order). Likewise, the value of CR is ≥0.70, and VE is ≥0.50. Thus, it can be concluded that all the indicators used in this study have good validity and reliability values.

**Table 6:** Initial Model Values, Modified Model Values, and One Factor Model Values of EL, CA, and PWB

Construct	λ	Initial Model Values							Modified Model Values						One Factor Model Values					
		λ^2^	E	α	MaxR (H)	CR	AVE	Sqrt AVE	λ	λ^2^	E	CR	AVE	Sqrt AVE	λ	λ^2^	E	CR	AVE	Sqrt AVE
EL	0.476	0.23	0.77	**0.827**					0.478	0.23	0.77				0.472	0.22	0.78	**0.871**	**0.501**	**0.708**
	0.801	0.64	0.36						0.828	0.69	0.31				0.803	0.64	0.36			
	0.493	0.24	0.76						0.471	0.22	0.78				0.469	0.22	0.78			
	0.579	0.34	0.66		**0.900**	**0.860**	**0.478**	**0.692**	0.684	0.47	0.53	**0.870**	**0.499**	**0.707**	0.722	0.52	0.48			
	0.771	0.59	0.41						0.762	0.58	0.42				0.763	0.58	0.42			
	0.836	0.70	0.30						0.842	0.71	0.29				0.842	0.71	0.29			
	0.78	0.61	0.39						0.776	0.60	0.40				0.781	0.61	0.39			
CA	0.783	0.61	0.39	**0.843**					0.785	0.62	0.38				0.761	0.58	0.42	**0.826**	**0.552**	**0.743**
	0.663	0.44	0.56						0.595	0.35	0.65				0.584	0.34	0.66			
	0.885	0.78	0.22		**0.890**	**0.842**	**0.575**	**0.758**	0.926	0.86	0.14	**0.828**	**0.554**	**0.744**	0.953	0.91	0.09			
	0.681	0.46	0.54						0.622	0.39	0.61				0.615	0.38	0.62			
PWB	0.869	0.76	0.24	**0.869**					0.868	0.75	0.25				0.872	0.76	0.24	**0.883**	**0.654**	**0.809**
	0.758	0.57	0.43		**0.892**	**0.883**	**0.655**	**0.809**	0.757	0.57	0.43	**0.883**	**0.655**	**0.809**	0.755	0.57	0.43			
	0.841	0.71	0.29						0.84	0.71	0.29				0.843	0.71	0.29			
	0.764	0.58	0.42						0.767	0.59	0.41				0.759	0.58	0.42			
SRMR = 0.092									**SRMR = 0.090**						**SRMR = 0.0795** **EL**: **SRMR = 0.037** **CA: SRMR = 0.011** **PWB: SRMR = 0.008**					

MSV is the square of the highest correlation coefficient between latent constructs MSV = 0.239

ASV is the mean of the squared correlation coefficients between latent constructs. ASV = 0.171

EL < −-> PWB Correlation: 0.489 MSV:0.239

CA < −-> PWB Correlation: 0.362 MSV:0.131

EL < −-> CA Correlation: 0.377 MSV: 0.142

## Discussion

Healthcare institutions are undergoing a fast transformation in response to a variety of difficulties, including enhancing public health, generating new health concerns, applying new information, and requiring cost-effective therapies. Entrepreneurial leaders are necessary for success in this globalized society. Considering technical advancement, the economic crisis, and a volatile marketplace, an entrepreneurial leader ought to steer the firm in an upward trajectory. Additionally, the entrepreneurial leader creates possibilities, gives individuals power, and upholds connections inside the company to enhance achievement [37, 38].

The current study reported that nurses reported a moderate mean EL score in this regard. Possibly the nurse managers could empower nurses to better manage health services and ensure that goals and objectives are met regularly and high-quality services are provided to patients. Slowly, they can accomplish challenging tasks through innovative means toward the organizational vision. Further, they believe that their leaders work hard to find ways to raise the performance of nurses by appealing to their needs and the requirements of patients. The present findings aligned with the findings of Zare et al. (2022). On the flip hand, conflicting findings were revealed by Wardan (2020) and da Paixão Silva et al. (2017), who stated that most nurse managers lack entrepreneurial traits. Also, studies conducted by Afsar et al. (2016), Sarwoko (2020), Jakobsen et al. (2021), Zhang (2021), and Sarnkhaowkhom et al. (2022) show that many nurses in managerial roles typically exhibit EL at a high level, which might be described as the nursing profession’s version of an entrepreneur [39–46].

The perception of moderate frequency of PWB by nurses was clarified by Abbas Khan (2021), and Matsuo et al. (2021) [47, 48]. This finding is consistent with the study’s findings, and it may be attributed to the nurses’ lack of initiative in thinking through, planning, and acting to address the problem as it exists. In addition, restrict their capacity to develop novel methods, look for root causes of issues, streamline processes, and communicate concepts. Additionally, it diminished their capacity to come up with novel solutions that stop issues from happening again. They were also unable to assume leadership roles in certain aspects of the job, including missions of the hospitals, standards for nursing, guidelines, and quality assurance.

This contrasts with Molin et al.’s (2019) findings, which show that nurses in management positions exhibit more proactive behavior. These nurses are accountable for leading or developing change proposals and coordinating teams to accomplish the suggested objectives. However, Frögéli (2019) discussed how avoiding PWB helps newly registered nurses cope with the symptoms of stress-related illness and helps them transition to their new role in the workplace [49, 50].

The current study’s findings regarding CA showed that nurses thought their level of adaptability was moderate. This outcome may be the result of the complicated and dynamic character of the healthcare industry, as well as the fact that high workloads have limited nurses’ capacity to respond to stress related to current and future career growth and deal with unforeseen circumstances and job circumstances.

It also prevents them from making judgments in a clear and timely manner in reaction to changes and from quickly adjusting to the workplace atmosphere. Several studies by Dateling (2021), Chen & Zhang (2023), and Zhang et al. (2023) that found a moderate level of CA among nurses corroborated the findings of this study. Additionally, average CA perception among nurses was discovered by Kwak & Kwon (2017), Kim & Woo (2018), Lee & Lee (2019), and Jahani et al. (2022) [29, 51–56].

A structural equation model was created to test this research hypothesis. The model’s results supported H1, which states that EL has a significant relationship with nurses’ PWB and CA and shows a statistically significant positive correlation between the variables under study. This may be explained by the fact that ambitious nurse entrepreneurs are more likely to have a strong desire to adapt to their careers and impart these abilities to others who follow them. Additionally, given their propensity to recognize and seize professional possibilities as well as to design work settings that align with their interests, they are well-prepared for changes that may be pertinent to their line of work.

Furthermore, EL significantly affects an organization in several aspects, including growth, profitability, creativity, and innovation. Nurse Managers are encouraged by EL to be flexible in their adaptation to an unpredictable environment; this requires being creative and prepared to take chances to change one’s behavior, create new values, and take advantage of opportunities. Improve their capacity to handle both the anticipated duties of getting ready for and performing the job as well as the unforeseen alterations brought on by shifting work and working environment.

The findings of Cai et al. (2019), Nurjaman et al. (2019), Newman et al. (2018), Yang et al. (2019), Chebbi et al. (2020), Pauceanu et al. (2021), Škare et al. (2022), and Wahab & Tyasari (2020) substantiate this. These studies show that an entrepreneurial leader fosters a culture of exploration and exploitation by instilling confidence in their subordinates [14, 57–63]. Moreover, it was made evident by Tolentino et al. (2014), McKenna et al. (2016), and Qiao & Huang (2019) that there was a favorable correlation between entrepreneurial leadership and a high level of CA among nurses. Abbas (2022) as well as Simba and Thai (2018) concluded, that EL promotes PWB, opportunity finding, inventive thinking, and taking measured risks [64–68]..

## Conclusion

The results imply and support the statistically significant relationship between EL and the PWB and the CA of nurses. This study has multiple theoretical contributions based on the findings presented in the present study. First, this research adds to the literature on entrepreneurial leadership by developing and testing a new model through which entrepreneurial leadership promotes nurses’ PWB and CA. Moreover, the findings of this research have wide-ranging implications for business leaders and entrepreneurs, both existing and emerging, who ought to encourage PWB and CA among their employees to maximize the growth and competitiveness of their organizations in the long term.

### Limitations of the study

The limitations of the present research provide opportunities for further studies in the area. First, we concentrated on developing a structure equation model of EL, nurses’ PWB, and CA, and the limitations of this study can be illustrated as follows; the data was collected from only two private hospitals thus the generalization of findings might be restricted. Second, even though this study employed the SEM approach, the cross-sectional study design makes it impossible to demonstrate causality, which limits the evaluation of the influence of study factors. Finally, the data was collected through self-report measures that may be sensitive to subjectivity and response bias. To address these limitations, objective metrics will be required in the future through observational, longitudinal, qualitative, empirical, and multi-site research.

### Implications

The research on the influence mechanism between EL and PWB and career adaptability has theoretically been enhanced and reinforced by this study. The practical ramifications of this study extend to staff nurses, nurse managers, and hospital human resources management practices. First, this study contributes to the body of knowledge on EL by creating and evaluating a novel model that explains how nurse managers’ EL fosters PWB and CA in nurses while also illuminating the relationship between these factors.

Second, there aren’t any studies evaluating the impact of EL on nurses’ PWB and CA that we are aware of in entrepreneurship literature. Additionally, this study offers novel insight that shows how entrepreneurial leaders enable staff members to create successful outcomes for nurses. Leaders can also utilize the study’s results to support EL as a means of fostering proactivity and CA. Thus, to enhance nurses’ PWB and CA as a proactive strategy for upgrading career success and outstanding organizational objectives and staying on top of the rapidly evolving healthcare industry, it is necessary to continuously update job knowledge and skills through additional training and educational programs.

Furthermore, managers can utilize the study’s results to support entrepreneurial leadership in fostering proactive environments that empower staff members to take initiative, feel self-assured, and develop their career flexibility. Also, these results inspire managers to create new healthcare business chances, and detecting market needs is a fundamental aspect of EL. Structuring a strategic, operational, and sustainable healthcare business plan that will be implemented by entrepreneurial leaders and proactive staff who are adaptable to their career opportunities. Additionally, hospital managers should recruit and retain top-talent staff to build EL with high-performing teams with enduring development opportunities. The research’s conclusions can also be used by academics through studying entrepreneurship in more research methodologies to better comprehend the additional tasks and duties that contemporary and aspiring business leaders must fulfill as well as to assist them hone their entrepreneurial leadership competencies.

## Data Availability

No datasets were generated or analysed during the current study.

## Abbreviations

EL: Entrepreneurial leadership
PWB: Proactive work behavior
CA: Career adaptability
SCT: Social cognitive theory

## References

[1] D’Souza J (2023). Entrepreneurial leadership strategies for catalyzing innovation performance.

[2] Agung A, Landra N, Sudja N (2020). Role of ethical behavior and entrepreneurial leadership to improve organizational performance. Cogent Bus Manag.

[3] Pajic S, Keszler Á, Kismihók G, Mol ST, Den Hartog DN (2018). Antecedents and outcomes of Hungarian nurses’ career adaptability. Int J Manpow.

[4] Mohamed Al Anwer Ashour H, Diab Ghanem Atalla A (2021). The relationship between entrepreneurship education, entrepreneurial intention and career aspiration among faculty of nursing students. Egypt J Nurs Health Sci.

[5] Bandura A (1988). Organizational applications of social cognitive theory. Aust J Manag.

[6] Pu B, Sang W, Yang J, Ji S, Tang Z (2022). The effect of entrepreneurial leadership on employees’ tacit knowledge sharing in start-ups: a moderated mediation model. Psychol Res Behav Manag.

[7] Renko M, El Tarabishy A, Carsrud AL, Brännback M (2015). Understanding and measuring entrepreneurial leadership style. J Small Bus Manag.

[8] Bagheri A, Akbari M (2018). The impact of entrepreneurial leadership on nurses’ innovation behavior. J Nurs Scholarsh.

[9] Naushad M (2021). Investigating determinants of entrepreneurial leadership among smes and their role in sustainable economic development of Saudi Arabia. J Asian Finance Econ Bus.

[10] Bagheri A, Harrison C (2020). Entrepreneurial leadership measurement: a multidimensional construct. J Small Bus Enterp Dev.

[11] Lin Q, Yi L. The multilevel effectiveness of entrepreneurial leadership: a meta-analysis. J Manag Organ. 2021:1–19. 10.1017/jmo.2020.45.

[12] Ersarı G, Naktiyok A (2022). The role of competitive strategies in the effect of entrepreneurial mindset and entrepreneurial leadership on business performance. Ist Bus Res.

[13] Li X (2020). The preliminary literature review of proactive behavior. Am J Ind Bus Manag.

[14] Nurjaman K, Marta MS, Eliyana A, Kurniasari DR, Kurniasari D (2019). Proactive work behavior and innovative work behavior: moderating effect of job characteristics. Hum Soc Sci Rev.

[15] Kim JE (2021). Paradoxical leadership and proactive work behavior: the role of psychological safety in the hotel industry. J Asian Finance Econ Bus.

[16] Al-Fatlawi HB, Amanah AA (2021). Effect of adopting proactive work behaviors on achieving strategic entrepreneurship. PalArch's J Archaeol Egypt/ Egyptol.

[17] Permata FD, Mangundjaya WL (2021). The role of work engagement in the relationship of job autonomy and proactive work behavior for organizational sustainability. IOP Conf Ser: Earth Environ Sci.

[18] Hu X, He Y, Ma D, Zhao S, Xiong H, Wan G (2021). Mediating model of college students’ proactive personality and career adaptability. Career Dev Q.

[19] Wen Y, Liu F, Pang L, Chen H (2022). Proactive personality and career adaptability of Chinese female pre-service teachers in primary schools: the role of calling. Sustain.

[20] Sun C, Xing Y, Wen Y, Wan X, Ding Y, Cui Y, Xu W, Wang X, Xia H, Zhang Q, Yuan M (2023). Association between career adaptability and turnover intention among nursing assistants: the mediating role of psychological capital. BMC Nurs.

[21] Xiao Y, He Y, Gao X, Lu L, Yu X (2021). Career exploration and college students’ career adaptability: the mediating role of future work self-salience and moderating role of perceived teacher support. Discret Dyn Nat Soc.

[22] Porfeli EJ, Savickas ML (2012). Career adapt-abilities scale-USA form: psychometric properties and relation to vocational identity. J Vocat Behav.

[23] Obschonka M, Hahn E, Bajwa NH (2018). Personal agency in newly arrived refugees: the role of personality, entrepreneurial cognitions and intentions, and career adaptability. J Vocat Behav.

[24] Stead GB, LaVeck LM, Rúa SMH (2022). Career adaptability and career decision self-efficacy: Meta-analysis. J Career Dev.

[25] Ali Awad NH, Al-anwer Ashour HM (2022). Crisis, ethical leadership and moral courage: ethical climate during COVID-19. Nurs Ethics.

[26] Buchan J, Catton H. Recover to rebuild: investing in the nursing workforce for health system effectiveness. International Council of Nurses. 2023; Accessed Apr 11, 2023. https://www.icn.ch/system/files/2023-03/ICN_Recover-to-Rebuild_report_EN.pdf

[27] Bilal M, Chaudhry S, Amber H, Shahid M, Aslam S, Shahzad K (2021). Entrepreneurial leadership and employees’ proactive behaviour: fortifying self-determination theory. J Open Innov: Technol Mark Complex.

[28] Wang X, Niu J, Dai Q, Liu M (2023). Effects of career adaptability and self-efficacy on transition shock among newly graduated nurses during the COVID-19 pandemic: a survey conducted in China. J Contin Educ Nurs.

[29] Jahani D, Jabbarzadeh Tabrizi F, Dadashzadeh A, Sarbakhsh P, Hosseinzadeh M (2022). Career adaptability and its correlation with quality of work life in nurses working in the emergency department. Saf Health Work.

[30] Htet HY, Abhicharttibutra K, Wichaikum OA. Factors predicting proactive work behaviors among nurses: A descriptive predictive study. Int Nurs Rev. 2023; 10.1111/inr.12856. 10.1111/inr.12856 37302103

[31] Malibari MA, Bajaba S (2022). Entrepreneurial leadership and employees’ innovative behavior: a sequential mediation analysis of innovation climate and employees’ intellectual agility. J Innov Knowl.

[32] Akeel AF, Elghannam HM, Abd El Fattah AM (2023). Entrepreneurial leadership and work engagement among nurse managers. Egyptian Nurs Health Sci.

[33] Parker SK, Collins CG (2010). Taking stock: integrating and differentiating multiple proactive behaviors. J Manag.

[34] Bilal M, Chaudhry SA, Sharif I, Shafique O, Shahzad K (2022). Entrepreneurial leadership and employee wellbeing during COVID-19 crisis: a dual mechanism perspective. Front Psychol.

[35] Işık E, Yeğin F, Koyuncu S, Eser A, Çömlekciler F, Yıldırım K (2018). Validation of the career adapt-abilities scale–short form across different age groups in the Turkish context. Int J Educ Vocat Guid.

[36] Schermelleh-Engel K, Moosbrugger H, Müller H (2003). Evaluating the fit of structural equation models: tests of significance and descriptive goodness-of-fit measures. Methods Psychol Res Online.

[37] Specchia ML, Cozzolino MR, Carini E, Di Pilla A, Galletti C, Ricciardi W, Damiani G (2021). Leadership styles and nurses’ job satisfaction results of a systematic review. Int J Environ Res Public Health.

[38] Suswati E, Gajayana MU (2022). Entrepreneurial leadership and learning organization its influence on midwives’ performance mediating by organizational commitment. J Entrep.

[39] Zare Qala Seyyedi F, Havasi B, Fadaei DN (2022). The relationship between leadership style and organizational entrepreneurship in the staff managers of Ahvaz Jundishapur University of Medical Sciences. Evid Based Health Policy Manag Econ.

[40] Wardan S, Ghandour S, Elghabbour MG (2020). Entrepreneurship and work innovation among nurse managers at Port Said governmental hospitals. Port Said Sci Jour of Nur.

[41] Da Paixão Silva AC, CavalcantiValente GL, Cavalcanti Valente GS (2017). Entrepreneurship as a tool for the nurse’s work. J Nurs UFPE online.

[42] Afsar B, Badir YF, Saeed BB, Hafeez S (2016). Transformational and transactional leadership and employee’s entrepreneurial behavior in knowledge–intensive industries. Int J Hum Resour Manag.

[43] Sarwoko E (2020). Entrepreneurial leadership and innovative work behavior: the role of creative self-efficacy. J Econ Bus Acc Vent.

[44] Jakobsen L, Wacher Qvistgaard L, Trettin B, Juel RM (2021). Entrepreneurship and nurse entrepreneurs lead the way to the development of nurses' role and professional identity in clinical practice: a qualitative study. J Adv Nurs.

[45] Zhang X. Entrepreneurial leadership to foster innovative output via psychological empowerment: role modeling is not enough. Dep Hum Res Org Beh. 2021:16–22. https://repositorio.iscte-iul.pt/bitstream/10071/23727/1/master_xinyi_zhang.pdf

[46] Sarnkhaowkhom C, Santre S, Phonsuk P, Wongtawee N, Piansamer S, Laohapisitpanich A, Suriyalerd W, Supapote N, Kaewmuean T, Hosangon N, Mathaworn S, Phikunthong P (2022). Assessment of entrepreneurial leadership among undergraduate nursing students: The case from Thailand. Nurse Media J Nurs.

[47] Abbas KN (2021). Determinants of proactive work behavior of employees during the COVID-19 crisis: a perspective on toxic leadership in the virtual work setting. Eur J Psychol Open.

[48] Matsuo M, Matsuo T, Arai K (2021). The influence of an interactive use of management control on individual performance: mediating roles of psychological empowerment and proactive behavior. J Account Organ Chang.

[49] Molin TD, Oliveira JLC, Tonini NS, Oliveira RM, Souza RF, Wawzeniak de Anchieta D, et al. Proactive behavior of hospital nurses: comparison between jobs. Cogitare Enferm. 2019;24 10.5380/ce.v24i0.58174.

[50] Frögéli E (2019). Testing principles from cognitive behavior therapy for preventing stress-related ill health among newly registered nurses (Order No. 28423475).

[51] Dateling TR (2021). The moderating effect of cultural values on career adaptability and work engagement (Order No. 28821066).

[52] Chen J, Zhang X (2023). The impact of career calling on higher vocational nursing students' learning engagement: the mediating roles of career adaptability and career commitment. Front Psychol.

[53] Zhang J, Zhao C, Li F, Wang X, Xu H, Zhou M, Huang Y, Yang Y, Yu G, Zhang G (2023). Longitudinal relationships among career adaptability, resilience, and career commitment in Chinese nursing undergraduates: testing differences in career interest between cross-lagged models. BMC Nurs.

[54] Kwak MH, Kwon SB (2017). Professional self-concept and organizational socialization of new nurse. J Korea Converg Soc.

[55] Kim NY, Woo CH (2018). Mediating effect of self-efficacy in the relationship between informal learning, shared leadership and organizational socialization of beginner advanced beginner nurses. J Korean Acad Nurs Adm.

[56] Lee KH, Lee MJ (2022). The effect of new nurses’ clinical competence on career adaptation. Annals of R.S.C.B.

[57] Cai W, Lysova EI, Khapova SN, Bossink BAG (2019). Does entrepreneurial leadership Foster creativity among employees and teams? The mediating role of creative efficacy beliefs. J Bus Psychol.

[58] Newman A, Herman H, Schwarz G, Nielsen I (2018). The effects of employees’ creative self-efficacy on innovative behavior: the role of entrepreneurial leadership. J Bus Res.

[59] Yang J, Pu B, Guan ZZ (2019). Entrepreneurial leadership and turnover intention of employees: the role of affective commitment and person-job fit. Int J Environ Res Public Health.

[60] Chebbi H, Yahiaoui D, Sellami M, Papasolomou I, Melanthiou Y (2020). Focusing on internal stakeholders to enable the implementation of organizational change towards corporate entrepreneurship: a case study from France. J Bus Res.

[61] Pauceanu AM, Rabie N, Moustafa A, Jiroveanu DC (2021). Entrepreneurial leadership and sustainable development—a systematic literature review. Sustain.

[62] Škare M, Blanco-Gonzalez-Tejero C, Crecente F, Del Val MT (2022). Scientometric analysis on entrepreneurial skills - creativity, communication, leadership: how strong is the association?. Technol Forecast Soc Chang.

[63] Wahab A, Tyasari I (2020). Entrepreneurial leadership for university leaders: a futuristic approach for Pakistani HEIs. Asia Pac Manag Rev.

[64] Tolentino LR, Garcia M, Lu VN, Restubog SLD, Bordia P, Plewa C (2014). Career adaptation: the relation of adaptability to goal orientation, proactive personality, and career optimism. J Vocat Behav.

[65] McKenna B, Zacher H, Sattari Ardabili F, Mohebbi H (2016). Career adapt-abilities scale–Iran form: psychometric properties and relationships with career satisfaction and entrepreneurial intentions. J Vocat Behav.

[66] Qiao XP, Huang JH (2019). Effect of college students’ entrepreneurial self-efficacy on entrepreneurial intention: career adaptability as a mediating variable. Int J Educ Methodol.

[67] Abbas AA (2022). The role of organizational virtuousness in reinforcement proactive work behavior. Manage Org: Syst Res.

[68] Simba A, Thai MTT (2019). Advancing entrepreneurial leadership as a practice in MSME management and development. J Small Bus Manag.

